# Resection of anorectal fistula cancer associated with Crohn’s disease after preoperative chemoradiotherapy: a case report

**DOI:** 10.1186/s40792-023-01778-6

**Published:** 2023-11-14

**Authors:** Takuya Inoue, Yuki Sekido, Takayuki Ogino, Tsuyoshi Hata, Norikatsu Miyoshi, Hidekazu Takahashi, Mamoru Uemura, Tsunekazu Mizushima, Yuichiro Doki, Hidetoshi Eguchi

**Affiliations:** 1 Department of Gastroenterological Surgery, Graduate School of Medicine, Osaka University, 2-2 Yamadaoka E-2, Suita, Osaka 565-0871 Japan; 2 Department of Gastroenterological Surgery, Osaka Police Hospital, Tennoji-Ku Kitayamacho 10-31, Osaka City, Osaka, 543-0035 Japan

**Keywords:** Anorectal fistula cancer, Chemoradiotherapy, Total perineal exenteration, Crohn’s disease

## Abstract

**Background:**

Anorectal fistula cancer is often diagnosed in an advanced state, and radical resection is difficult when invasion of the pelvic wall is observed. In addition, there is currently no clear evidence for perioperative treatment of locally advanced cases. We report a case of anorectal fistula cancer with widespread infiltration diagnosed during the course of Crohn’s disease, which was curatively resected after preoperative chemoradiotherapy.

**Case presentation:**

A 49-year-old man who had been diagnosed with Crohn’s disease (ileocolonic type) at the age of 25 and was found to have an anorectal fistula and perianal abscess at the age of 44 was referred to our department with complaints of abdominal pain and diarrhea. Computed tomography (CT) showed anal stenosis due to a pelvic mass. Pathological analysis of a biopsy taken under general anesthesia indicated mucinous carcinoma. Magnetic resonance imaging (MRI) revealed infiltration into the prostate, seminal vesicles, levator ani muscle, and left internal obturator muscle, and the patient was diagnosed with cT4N0M0 cStage IIIB anorectal fistula cancer (UICC TNM classification 8th edition). After performing a laparoscopic sigmoid colostomy, chemoradiation therapy (capecitabine + oxaliplatin, 50.4 Gy/28fr) was initiated. The patient then underwent laparoscopic total pelvic exenteration, colonic conduit diversion, extensive perineal resection, and reconstruction using bilateral gluteus maximus flaps and a right rectus abdominis musculocutaneous flap. The pathological diagnosis was mucinous adenocarcinoma, pT4, and all margins were negative. No recurrence was evident 6 months after the operation without adjuvant chemotherapy.

**Conclusion:**

We described a case of curative resection after preoperative chemoradiotherapy for anorectal fistula cancer with extensive invasion that was diagnosed during the course of Crohn’s disease.An accumulation of cases is needed to determine the usefulness of preoperative chemoradiation therapy for local control of anorectal fistula cancer associated with Crohn’s disease.

## Background

Anal fistula is the most common anal complication of Crohn’s disease (CD) in Japan. Most anorectal fistula cancers are found because of changes in the clinical symptoms, such as mucus discharge, anal bleeding, and stricture that was not seen previously. As various clinical symptoms of anal fistula make early diagnosis difficult, anorectal fistula cancer is often diagnosed in an advanced state. Anorectal fistula cancer is relatively rare among anal canal cancers, and it has been reported to account for 6.9% of anal canal cancers in Japan [1]. Due to the rarity, there is currently no clear evidence for perioperative treatment of locally advanced cases.

Here, we report a case of anorectal fistula cancer with widespread infiltration diagnosed during the course of CD that was curatively resected after preoperative chemoradiotherapy.

## Case presentation

**Fig. 1:**
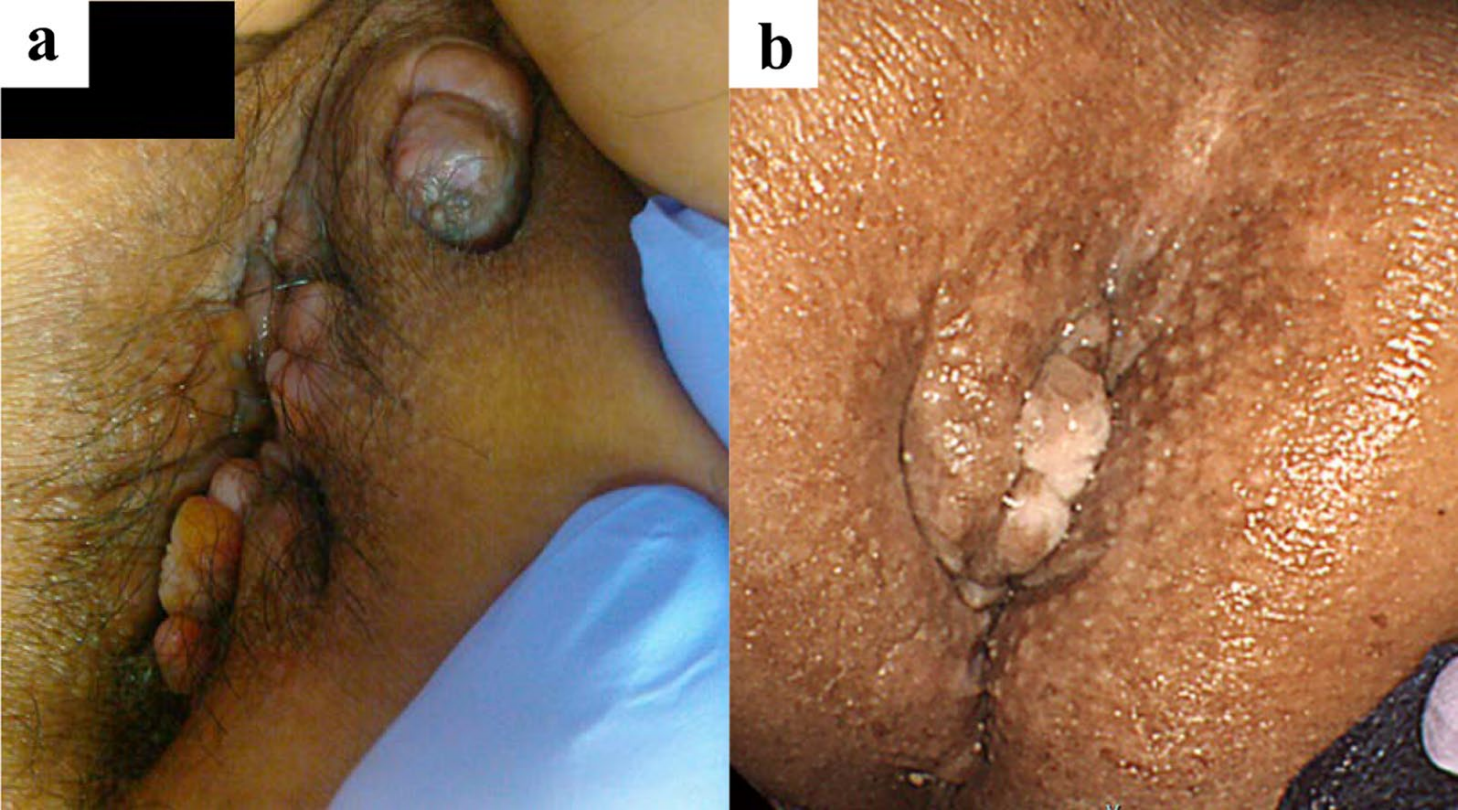
Preoperative macroscopic examination of the anus. **a** Initial anal findings showed a circumferential protruded lesion and stenosis of the anus. **b** After chemoradiotherapy, the protruding anal lesion had shrunk

**Fig. 2:**
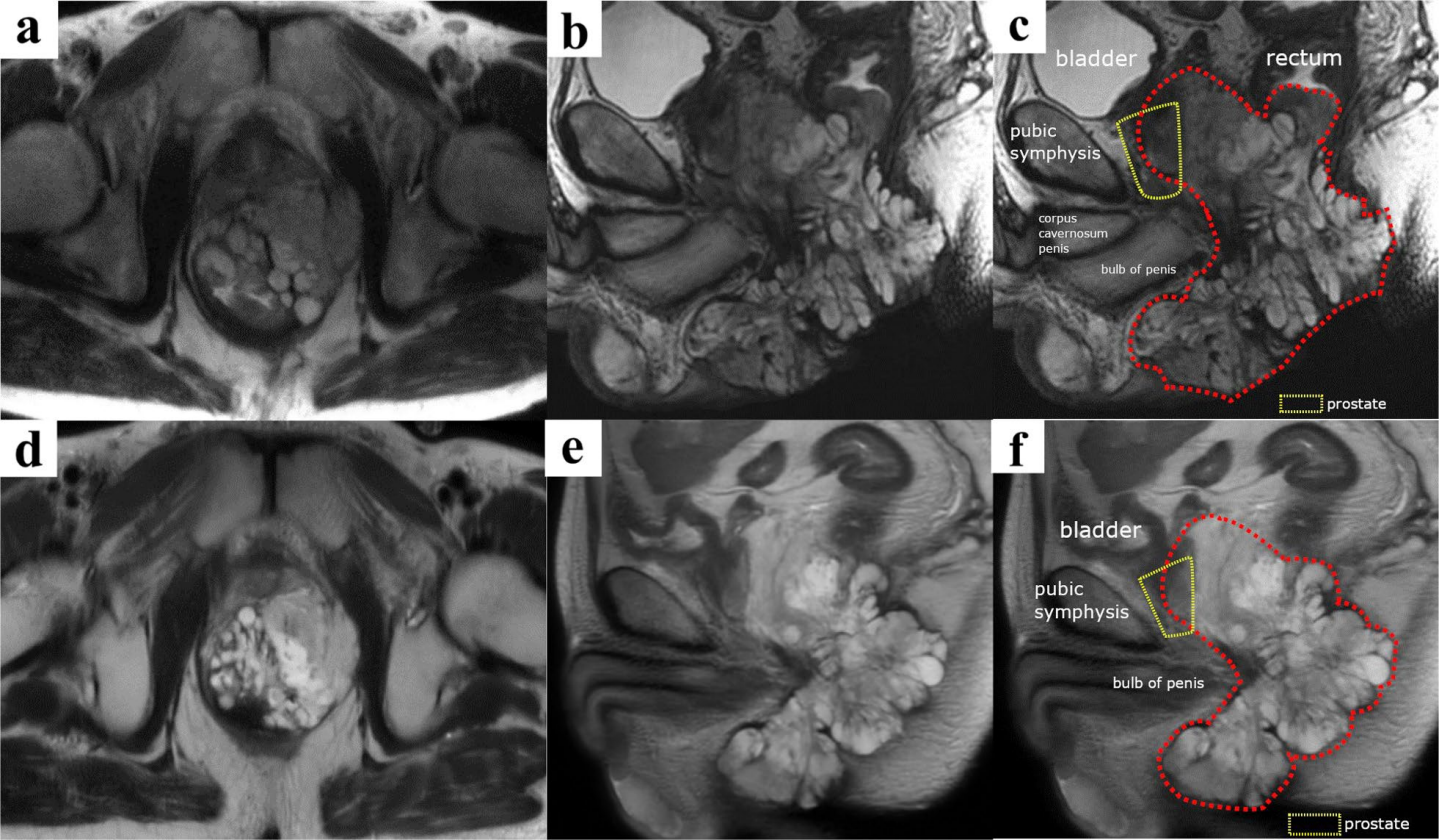
T2-weighted magnetic resonance imaging of the anorectal fistula cancer. Infiltration into the prostate, seminal vesicles, levator ani muscle, and left internal obturator muscle was revealed in the region surrounded by the red dotted line (**a**–**c**). After chemoradiotherapy, the tumor had slightly shrunk with stable disease as defined by the revised RECIST guidelines (version 1.1) (**d**–**f**)

**Fig. 3:**
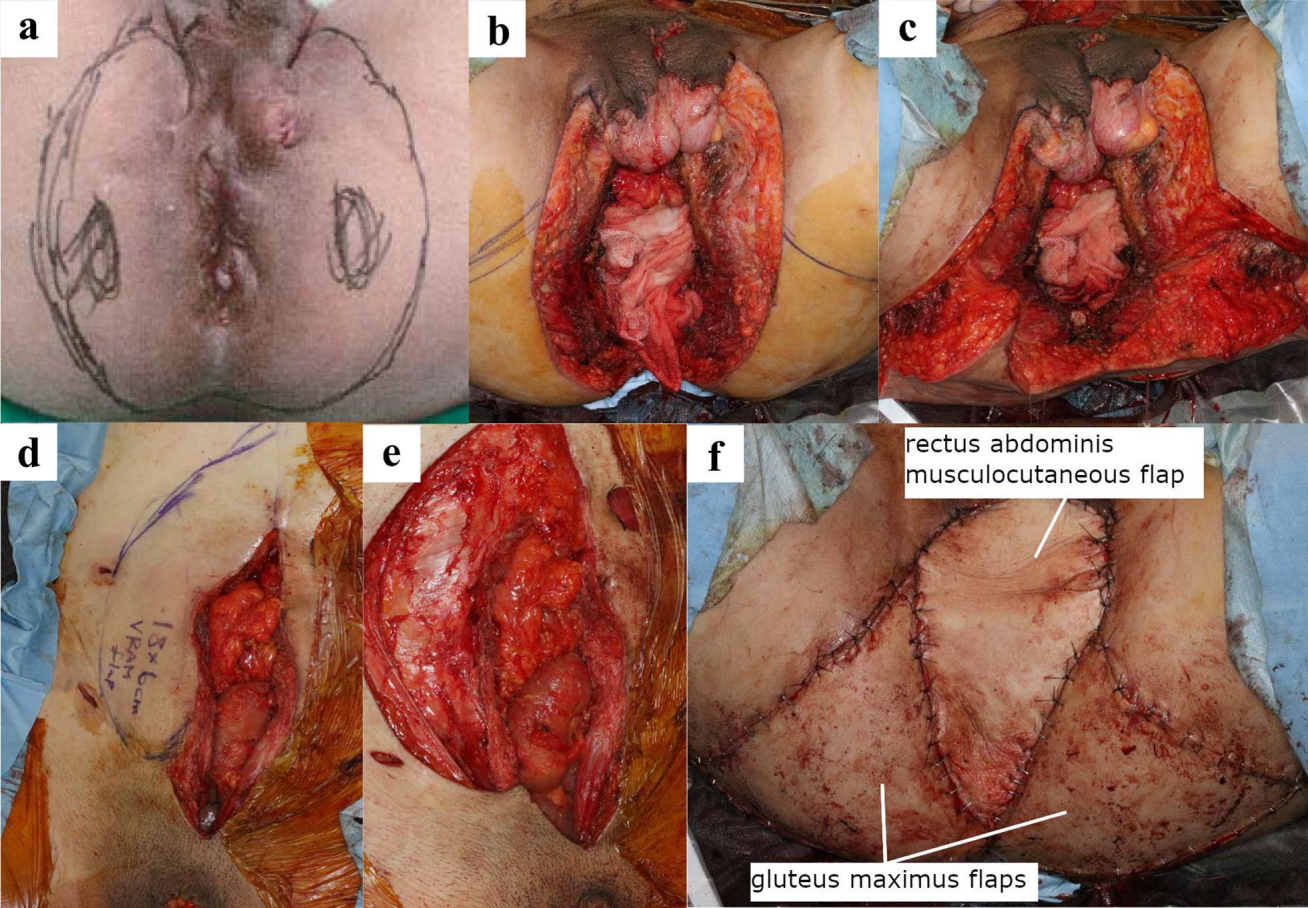
Intraoperative findings. We marked the resection line and exenterated the rectum, perianal skin, bladder, ureter, prostate, and penis en bloc (**a**, **b**), and then reconstructed using bilateral gluteus maximus flaps and a right rectus abdominis musculocutaneous flap (**c**–**f**)

**Fig. 4:**
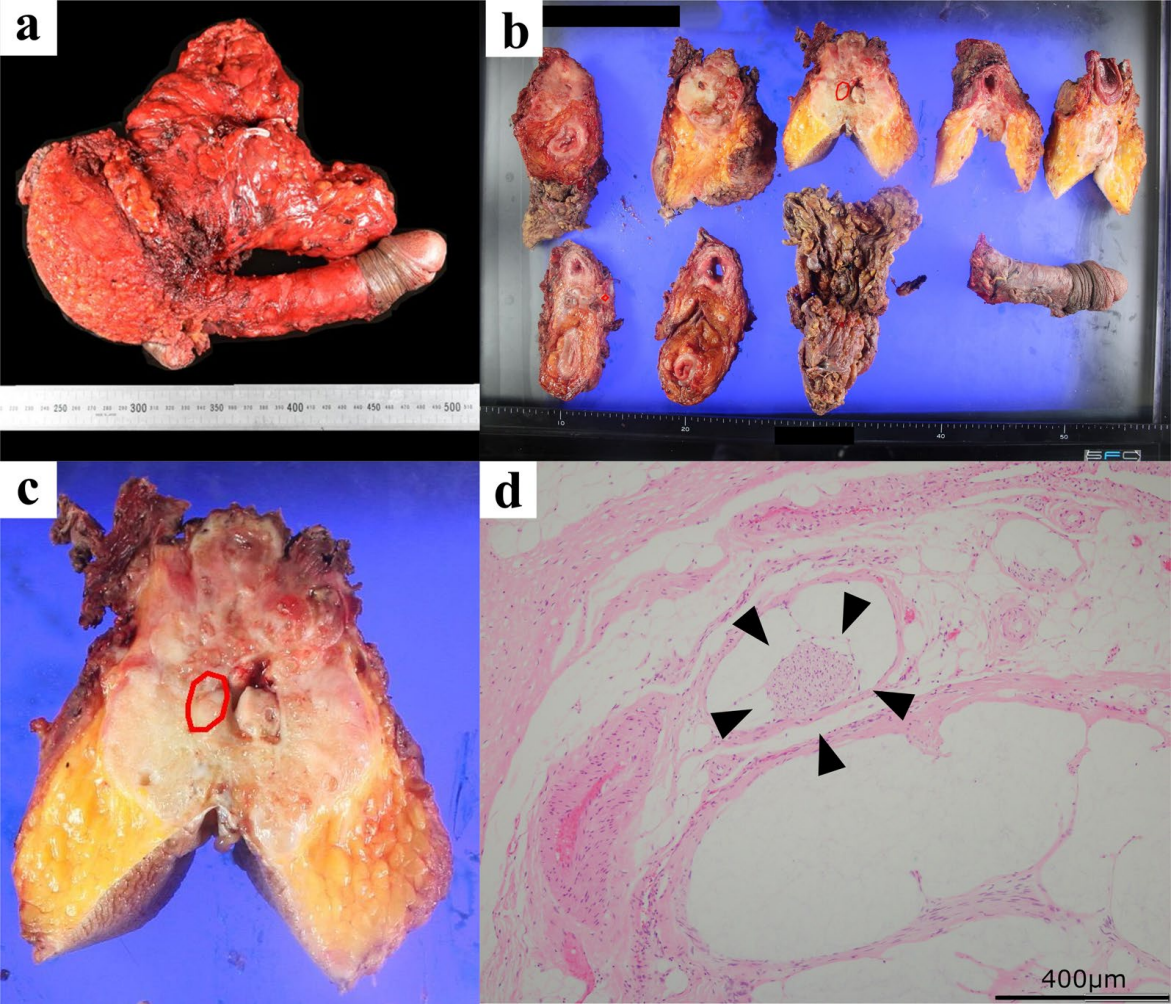
Gross and pathological findings of the specimen. Extensively infiltrated mucinous nodules were observed, mainly near the anus (**a**), but there were a few viable carcinomas only in the region of anal canal surrounded by the red line (**b**, **c**). Microscopic findings showed floating carcinomas indicated by the arrowheads within the mucinous nodules, which were markedly reduced compared to the biopsy findings (**d**)

A 49-year-old man was referred to our department with complaints of abdominal pain and diarrhea. He had been diagnosed with CD (ileocolonic type) at the age of 25 years. At 28 years old, he underwent small bowel resection for small bowel perforation and was introduced to azathioprine. He was found to have an anorectal fistula and perianal abscess at the age of 44 years. That same year, small bowel resection was performed for anastomotic stenosis and fistula formation, and infliximab was introduced during the postoperative period. The diagnosis according to the Montreal Classification was A2L3B2p. At our hospital, macroscopic examination found an entire circumferential protruded lesion and stenosis of the anus, so that the fifth finger was impassable (Fig. 1a). Computed tomography (CT) showed anal stenosis due to a pelvic mass. A percutaneous biopsy under local anesthesia failed to diagnose the disease; therefore, the patient underwent an examination under general anesthesia. Biopsy revealed a diagnosis of mucous carcinoma. Magnetic resonance imaging (MRI) suggested infiltration into the prostate, seminal vesicles, corpus cavernosum, levator ani muscle, and left internal obturator muscle (Fig. 2a). Laboratory studies showed no elevation of tumor markers CEA and CA19-9. Therefore, the clinical diagnosis was cT4N0M0, cStage IIIB anorectal fistula cancer according to the UICC TNM classification (8^th^ edition) [2]. Due to a rectal obstruction, laparoscopic sigmoid colostomy was performed before introducing neoadjuvant chemoradiotherapy (CRT) comprising 50.4 Gy in 28 fractions and intravenous oxaliplatin (130 mg/m^2^) on day 1 followed by oral capecitabine (1000 mg/m^2^) twice daily from the evening of day 1 to morning of day 15. The patient complained of diarrhea after one course of chemotherapy, so the regimen was changed to 5-fluorouracil + calcium levofolinate for the second course. Four weeks after completion of CRT, macroscopic examination found shrinkage of the protruding anal lesion (Fig. 1b). Follow-up CT and MRI revealed that the tumor had slightly shrunk with stable disease as defined by the revised Response Evaluation Criteria in Solid Tumors (RECIST) guidelines (version 1.1) [3]. Twelve weeks after CRT, we performed laparoscopic total pelvic exenteration, colonic conduit diversion, extensive perineal resection, and reconstruction using bilateral gluteus maximus flaps and a right rectus abdominis musculocutaneous flap. Because of CD, we did not use an ileal conduit, but instead created a colonic conduit with the remaining sigmoid colon. Due to infiltration into the corpus cavernosum, the penis was resected, but the testes were preserved. The rectum, perianal skin, bladder, ureter, prostate, and penis were exenterated en bloc. The abdominal wall defect was closed with interrupted sutures (Fig. 3). The operating time was 1104 min, and the blood loss volume was 580 mL. The postoperative course was complicated by infection of the abdominal wound. Otherwise, it was uneventful, and the patient was discharged on the 42nd postoperative day. Histopathological examination of the specimen showed mucinous adenocarcinoma, ypT4, INFb, Ly0, V0, Pn1b, RM0, N0 (Fig. 4). Infiltration of mucinous nodules into the prostate, seminal vesicles, corpus cavernosum, levator ani muscle, and left internal obturator muscle was detected, but there were no viable carcinomas in the region of invasion. The therapeutic effect was Grade 2 according to the Tumor Regression Grade [4]. Adjuvant chemotherapy was not introduced because there were no lymph node metastases and the resection margin was sufficient. No recurrence was evident 6 months after the operation.

## Discussion

Anorectal fistula cancer is defined by the WHO classification as a tumor arising from the anal sinus or fistula [5]. It is thought that anorectal fistula cancer develops from the anal glands within the anal fistula and the epithelial components of the fistula ducts, becoming malignant when stimulated by inflammation over a long period of time [6, 7]. In general, early diagnosis and treatment of anorectal fistula cancer is difficult due to the clinical symptoms caused by anal fistulas. A case has been reported in which a preoperative pathological diagnosis was not made despite multiple biopsies and a diagnosis of malignancy was finally obtained from the resected specimen [8]. In our case, although the patient had been followed up regularly for more than 5 years with anal stenosis due to anorectal fistula, routine endoscopy and biopsy failed to diagnose the anorectal fistula cancer, eventually revealed by a close examination for abdominal pain and diarrhea. Several researchers have reported case series and established the following five diagnostic criteria for anal fistula malignancy: repeated inflammation over a long period of time (> 10 years), pain and induration in the region of the anorectal fistula, mucinous discharge, no primary cancer in other parts of the anorectal region, and anorectal fistula orifice in the anal canal or anal crypt [9, 10].

Although anorectal fistula cancer is a rare disease in terms of colorectal cancer as a whole, anorectal fistula cancer is not uncommon in CD because an anal fistula is the most common anal lesion associated with CD in Japan [11]. It has been reported that 15% of colorectal cancers associated with CD are anorectal fistula cancers [12]. Early disease onset, long-standing disease (> 10 years), severe chronic colitis, chronic fistula, and stenosis are important risk factors for carcinogenesis of a fistula tract [13]. Colorectal cancer associated with CD is more common in the right side of the colon in Western countries and in the left side of the colon in Japan [11]. It is particularly common in the rectum and anus [14, 15], reportedly accounting for 55% of cases [12]. In Japan, the mean age at cancer diagnosis is 58.3 years for anorectal fistula cancer overall and 38.9 years for cancers associated with CD [1, 11], indicating that anorectal fistula cancer associated with CD tends to develop at a relatively young age. The duration from the onset of anorectal fistula to the diagnosis of cancer appears to be 18.8 years for all anorectal fistula cancers and 17 years for cases associated with CD [1, 11], which is not significantly different. In this patient, though it had been more than 20 years from the onset of CD to the diagnosis of cancer, it was only 5 years from the onset of anorectal fistula to the diagnosis of cancer, a relatively short period of time.

Surgical resection is the standard treatment for anorectal fistula cancer and abdominoperineal resection is commonly performed [16]. One-third of resected cases were reported to be positive for resection margins, indicating the difficulty of radical resection in anorectal fistula cancer [1]. As it is not easy to determine the range of tumor infiltration intraoperatively, it is necessary to carefully examine the region to be resected using various preoperative imaging techniques before planning the surgery and to perform a wide local resection to avoid positive margins. Mucinous carcinoma, the main histological type of hemorrhoidal carcinoma, is known to have low sensitivity to chemotherapy [17, 18], but there have been several reported cases in which curative resection was performed after preoperative CRT for anorectal fistula carcinoma. Positive resection margins were reported in 14% of patients who were treated with preoperative CRT, and preoperative CRT may be useful to ensure negative resection margins [19]. In our case, even though the tumor had slightly shrunk on MRI with stable disease as defined by the revised RECIST guidelines (version 1.1) [3], curative resection was performed with extensive resection including the penis. Given that the floating carcinomas within the mucinous nodules were markedly reduced compared to the biopsy findings, preoperative CRT may have contributed to the negative resection margins. No large-scale trials have examined the efficacy of preoperative CRT for anorectal fistula cancer, and there are many factors to be considered, such as irradiation modalities, irradiation range, and chemotherapy regimen. In our case, we introduced the regimen that we generally apply to locally advanced rectal cancer. Considering the local control effect of preoperative CRT for rectal cancer, preoperative CRT for anorectal fistula cancer is expected to improve the negative margins, local control, and prognosis.

## Conclusion

We reported a case of curative resection after preoperative chemoradiotherapy for anorectal fistula cancer with extensive invasion that was diagnosed during the course of CD. Surgical resection is the primary treatment for anorectal fistula cancer, but extensive resection is often required. In addition, positive resection margins and local recurrence are frequent. An accumulation of cases is needed to reveal the usefulness of preoperative CRT for local control of anorectal fistula cancer associated with CD.

## Data Availability

The data sets supporting the findings and inferences of this case report are included in this article.

## Abbreviations

CD: Crohn’s disease
CT: Computed tomography
MRI: Magnetic resonance imaging
CRT: Chemoradiotherapy
